# BHLHE40 Mediates Cross-Talk between Pathogenic T_H_17 Cells and Myeloid Cells during Experimental Autoimmune Encephalomyelitis

**DOI:** 10.4049/immunohorizons.2300042

**Published:** 2023-11-07

**Authors:** Melissa E. Cook, Irina Shchukina, Chih-Chung Lin, Tara R. Bradstreet, Elizabeth A. Schwarzkopf, Nicholas N. Jarjour, Ashlee M. Webber, Konstantin Zaitsev, Maxim N. Artyomov, Brian T. Edelson

**Affiliations:** Department of Pathology and Immunology, Washington University School of Medicine, St. Louis, MO

## Abstract

T_H_17 cells are implicated in the pathogenesis of multiple sclerosis and experimental autoimmune encephalomyelitis (EAE). We previously reported that the transcription factor basic helix-loop-helix family member e40 (BHLHE40) marks cytokine-producing pathogenic T_H_ cells during EAE, and that its expression in T cells is required for clinical disease. In this study, using dual reporter mice, we show BHLHE40 expression within T_H_1/17 and ex-T_H_17 cells following EAE induction. *Il17a*-Cre–mediated deletion of BHLHE40 in T_H_ cells led to less severe EAE with reduced T_H_ cell cytokine production. Characterization of the leukocytes in the CNS during EAE by single-cell RNA sequencing identified differences in the infiltrating myeloid cells when BHLHE40 was present or absent in T_H_17 cells. Our studies highlight the importance of BHLHE40 in promoting T_H_17 cell encephalitogenicity and instructing myeloid cell responses during active EAE.

## Introduction

The myelin oligodendrocyte glycoprotein (MOG)_35–55_ peptide–induced experimental autoimmune encephalomyelitis (EAE) animal model of multiple sclerosis in C57BL/6 mice represents a CD4^+^ T cell–mediated demyelinating disease of the CNS (1). Following immunization with MOG_35–55_ peptide and adjuvant, CD4^+^ T cells primed in the periphery develop into T_H_1 and T_H_17 cells that are both capable of mediating disease. Fate-reporter mice that make use of *Il17a*-driven Cre expression have been instrumental in identifying populations of CD4^+^ T cells that express both IL-17A and IFN-γ (here referred to as T_H_1/17 cells) as well as IFN-γ^+^ ex-T_H_17 cells following EAE induction (2), both of which have been associated with encephalitogenicity that is dependent on their GM-CSF production (2–6).

Both active immunization and adoptive transfer models of EAE have been useful for identifying important T cell–intrinsic regulators of neuroinflammation. Using adoptive transfer models of EAE, the transcription factor T-bet is needed for encephalitogenicity of both T_H_17 and so-called T_H_GM cells (CD4^+^ T cells cultured to enhance their GM-CSF production) (7–9); however, *Il17a*-Cre–mediated deletion of floxed alleles of either *Tbx21* (T-bet) or *Rorc* (RORγt) led to only modest reductions in active EAE clinical disease (10). Other transcription factors are important for T cell pathogenicity during actively induced EAE such as Fosl2 (11) and Blimp-1 (12), although these have not been selectively deleted in only T_H_17 cells to test their impact on clinical disease. Although a few transcription factors, such as JunB (13, 14) and STAT4 (15, 16), have been demonstrated as having important intrinsic roles in T_H_17 cells using *Il17a*-Cre–mediated deletion, other transcription factors also likely contribute to T_H_17 cell–intrinsic encephalitogenicity.

We and others have shown a cell-intrinsic requirement for the transcription factor basic helix-loop-helix family member e40 (BHLHE40) in CD4^+^ T cells for disease in actively induced EAE in *Bhlhe40*^−/−^ mice, through the action of BHLHE40 as a positive regulator of GM-CSF and negative regulator of IL-10 production (17–19). Furthermore, in an adoptive transfer model of EAE, BHLHE40-deficient MOG-specific T_H_1 or T_H_17 cells were incapable of mediating disease (20). Others have shown *Bhlhe40* induction downstream of the T_H_17 cell–associated transcription factors RORγt, RORα, and SATB1 (11, 21, 22). In the case of SATB1, overexpression of BHLHE40 restored GM-CSF production and pathogenicity by *Satb1*-deficient CD4^+^ T cells (22). Additionally, *Bhlhe40* expression has been correlated with a pathogenic CD4^+^ T cell transcriptional signature during EAE (23–26). In this study, we sought to more thoroughly explore the role of BHLHE40 specifically in T_H_17 cells during active EAE. Using a combination of BHLHE40 reporter and conditional knockout mice along with single-cell RNA sequencing (scRNA-seq), we identify specific roles for BHLHE40 in determining the pathogenicity of T_H_17 cells during EAE.

## Materials and Methods

### Mice

*Bhlhe40*^GFP^ (20) and *Bhlhe40*^fl/fl^ (27) mice have been previously described. *Bhlhe40*^GFP^ mice were crossed to *Il17a*-Cre (The Jackson Laboratory, 016879, *Il17a^tm1.1(icre)Stck^*/J) and *Rosa26*-TdTomato mice (The Jackson Laboratory, 007914, B6.Cg-*Gt(ROSA)26Sor^tm14(CAG-tdTomato)Hze^*/J). *Bhlhe40*^fl/fl^ mice were crossed to *Cd4*-Cre (022071, B6.Cg-Tg(Cd4-cre)1Cwi/BfluJ), *Il17a*-Cre, *Lyz2*-Cre (018956, B6N.129P2(B6)-*Lyz2^tm1(cre)Ifo^*/J), and *S100a8*-Cre mice (021614, B6.Cg-Tg(S100A8-cre,-EGFP)1Ilw/J) (all from The Jackson Laboratory). All mice were on the C57BL/6 background and used between 8 and 20 wk of age. Animal experiments were approved by the Animal Studies Committee of Washington University in St. Louis. Littermates were used when possible, and both male and female mice were used in experiments.

### Immunizations and induction of EAE

EAE induction, EAE clinical scoring, MOG_35–55_ peptide immunizations, and assessment of T cell responses in the draining lymph node (DLN) at day 7 postimmunization were performed as previously described (20). To achieve the best use of littermates and age-matched mice, independent experiments were performed and data were combined.

### Cell preparation and flow cytometry

These techniques have been previously described (20). In brief, DLNs were collected at day 7 postimmunization to prepare single-cell suspensions. Brains and spinal cords (CNS) were processed together from either naive or EAE-induced mice at day 14 postinduction. Surface staining and intracellular cytokine staining were performed using the Abs in Supplemental Table I. For scRNA-seq experiments, CD45^+^7-aminoactinomycin D^−^ live, single cells of the CNS were sorted using a FACS-Aria II (BD Biosciences), washed, and adjusted to 10^3^ cells/μl in PBS + 0.04% BSA.

### scRNA-seq and data analysis

scRNA-seq was performed at the McDonnell Genome Institute using the Chromium single cell 3’ library kit v2 and Chromium instrument (10x Genomics, Pleasanton, CA). Sequencing was performed on an Illumina HiSeq 4000 instrument. Data were processed as described (28) for dimensionality reduction, construction of t-distributed stochastic neighbor embedding (tSNE) plots, clustering, identification of cluster-specific genes, and differential expression analysis. Differentially expressed genes (adjusted *p* value <0.05 and log_2_ fold-change >0.35) were cross-referenced to the hallmark (29), Reactome (https://reactome.org/) (30), and KEGG (https://www.kegg.jp/) (31) gene sets in the Molecular Signatures Database (MSigDB) (https://gsea-msigdb.org/gsea/msigdb/index.jsp). Monocle 2 was used for pseudotemporal analysis of CNS myeloid cell populations (32, 33). Data have been deposited in the Gene Expression Omnibus (GSE234705).

### Statistical analysis

Data were analyzed with Prism (GraphPad Software) and one-way ANOVA, Student *t* tests, or Mann–Whitney *U* tests were used as indicated in individual figure legends. For relevant comparisons where no *p* value is shown, the *p* value was >0.05. Horizontal bars represent the means, and error bars represent the SEM. Although we did not directly account for interexperiment variability in experiments in which EAE disease scores were measured, we confirmed that trends were repeated in each independent experiment.

## Results

### BHLHE40 is expressed in T_H_1/17 and ex-T_H_17 cells

**FIGURE 1.**
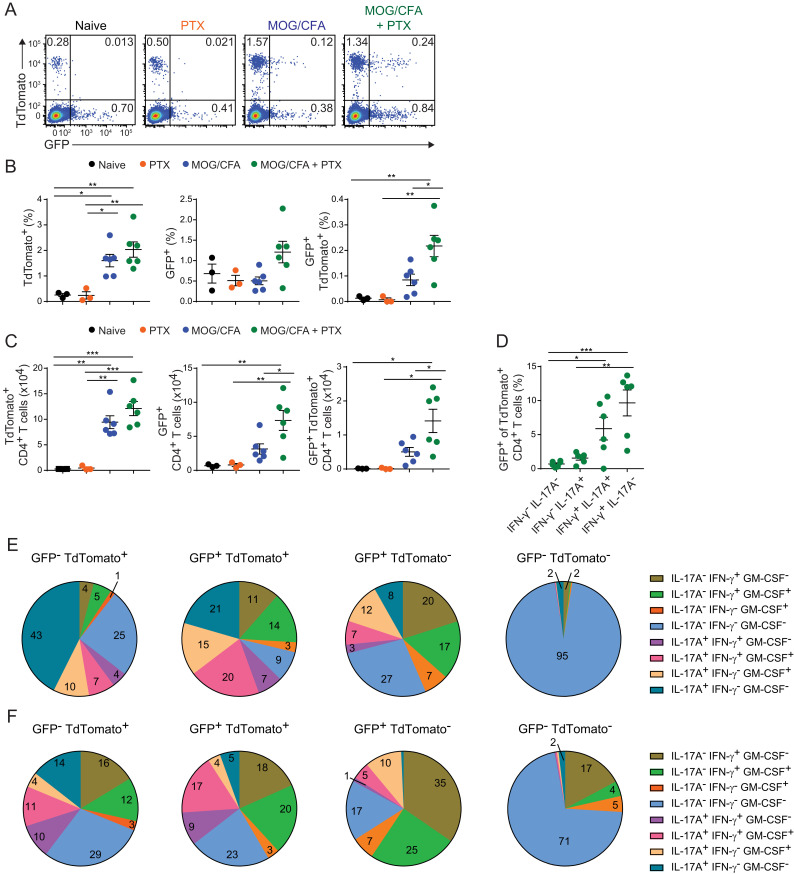
Expression of GFP in T_H_1/17 and ex-T_H_17 cells and cytokine production in double reporter mice. (**A**) Representative flow cytometry of TdTomato and GFP expression in CD4^+^ T cells (CD19^−^TCRβ^+^TCRγδ^−^CD4^+^CD8α^−^) isolated from DLNs of naive *Il17a*-Cre *Rosa26*-TdTomato *Bhlhe40*^GFP^ reporter mice or at day 7 postimmunization with pertussis toxin (PTX), MOG_35–55_ in CFA, or MOG_35–55_ in CFA plus PTX. (**B** and **C**) Percentages (B) or number (C) of TdTomato^+^, GFP^+^, or TdTomato^+^GFP^+^CD4^+^ T cells (pooled from two experiments, *n* = 3–6 per group). (**D**) The GFP^+^ percentage of TdTomato^+^CD4^+^ T cells at day 7 after MOG/CFA + PTX treatment within different cytokine-producing populations (pooled from two experiments, *n* = 6 per group). (**E**) Cytokine production from DLN CD4^+^ T cells from *Il17a*-Cre *Rosa26*-TdTomato *Bhlhe40*^GFP^ mice (pooled from two experiments, *n* = 4 per group) separated by TdTomato and GFP expression at day 7 after MOG/CFA + PTX treatment. (**F**) Cytokine production from CNS CD4^+^ T cells (CD45.2^+^CD19^−^TCRβ^+^TCRγδ^−^CD4^+^CD8α^−^) from *Il17a*-Cre *Rosa26*-TdTomato *Bhlhe40*^GFP^ mice (pooled from two experiments, *n* = 9 per group) separated by TdTomato and GFP expression at day 14 after MOG/CFA + PTX treatment. Data are presented as mean ± SEM. Statistical significance was determined by one-way ANOVA with a Tukey multiple comparison test (B, C, and D). *p < 0.05, **p < 0.01, ***p < 0.001.

To track BHLHE40 expression along with IL-17A fate mapping, we crossed *Bhlhe40*^GFP^ BAC transgenic mice to *Il17a*-Cre *Rosa26*-TdTomato mice. We immunized these mice with either pertussis toxin (PTX), MOG_35–55_ in CFA, or MOG_35–55_ in CFA with PTX injections, and on day 7 postimmunization, we analyzed the CD4^+^ T cell compartment for TdTomato and GFP (as a surrogate for BHLHE40) expression (Fig. 1A). Immunization with MOG_35–55_ in CFA increased the percentage and number of TdTomato^+^ CD4^+^ cells, but only with the addition of PTX was the percentage and number of GFP^+^ and GFP^+^ TdTomato^+^ CD4^+^ T cells increased (Fig. 1B, 1C). This is consistent with our previous finding that PTX stimulates secretion of IL-1β from myeloid cells, which induces BHLHE40 expression in CD4^+^ T cells (20). To ask whether BHLHE40 was expressed in T_H_1/17 and ex-T_H_17 cells, we examined TdTomato^+^CD4^+^ T cells for intracellular production of IFN-γ and IL-17A. GFP was highly expressed in IFN-γ^+^IL-17A^+^ T_H_1/17 cells and IFN-γ^+^IL-17A^−^ ex-T_H_17 cells (Fig. 1D).

We further characterized cytokine production of IFN-γ, IL-17A, and GM-CSF from the different types of reporter cells in the DLN. GM-CSF production correlated with GFP expression in both TdTomato^+^ and TdTomato^−^ CD4^+^ T cells (Fig. 1E). In addition, the greatest frequency of multifunctional CD4^+^ T cells (i.e., simultaneously producing IL-17A, IFN-γ, and GM-CSF) was found in the GFP^+^TdTomato^+^ group. A similar trend of cytokine production was seen in the CNS on day 14 after EAE induction (Fig. 1F, Supplemental Fig. 1). Within the CNS, a notable difference was an increase in IL-17A^−^IFN-γ^+^GM-CSF^−^ and IL-17A^−^IFN-γ^+^GM-CSF^+^ cells in both TdTomato^+^GFP^–^ ex-T_H_17 cells and TdTomato^−^GFP^−^ cells. This could indicate underreporting of BHLHE40 expression by the GFP reporter after permeabilization for intracellular cytokine staining or that some cells use BHLHE40-independent pathways to produce these cytokines. Nevertheless, the greatest fraction of total GM-CSF producers (i.e., IL-17A/IFN-γ agnostic) in both the DLN and CNS was found in GFP^+^ cells, consistent with the known role for BHLHE40 in supporting GM-CSF production.

### *Bhlhe40* deletion in IL-17A–expressing cells reduces neuroinflammation

**FIGURE 2.**
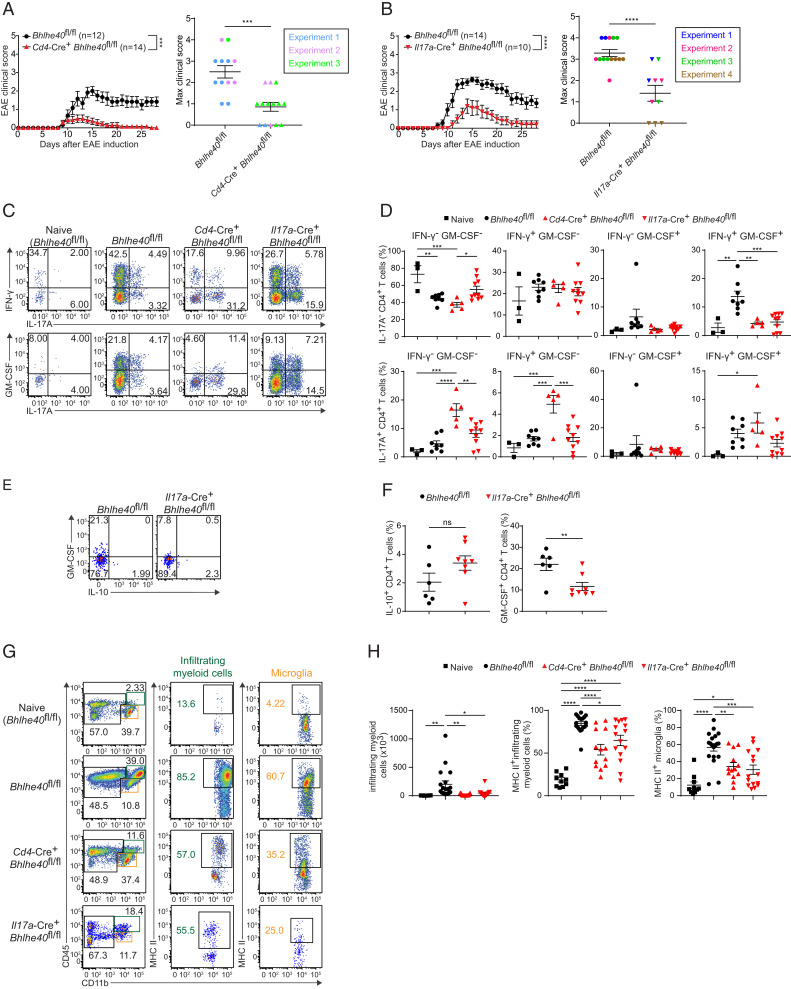
Decreased EAE severity in *Cd4*-Cre^+^ *Bhlhe40*^fl/fl^ and *Il17a*-Cre^+^ *Bhlhe40*^fl/fl^ mice. (**A**) Left, Clinical EAE scores from actively immunized *Cd4*-Cre^−^ *Bhlhe40*^fl/fl^ or *Cd4*-Cre^+^ *Bhlhe40*^fl/fl^ mice (pooled from three experiments, *n* = 12–14 per group). Right, Maximum clinical score of individual mice with independent experiments indicated by color. (**B**) Left, Clinical EAE scores from actively immunized *Il17a*-Cre^−^ *Bhlhe40*^fl/fl^ or *Il17a*-Cre^+^ *Bhlhe40*^fl/fl^ mice (pooled from four experiments, *n* = 10–14 per group). Right, Maximum clinical score of individual mice with independent experiments indicated by color. (**C**) Representative flow cytometry of cytokine production from CD4^+^ T cells (CD45.2^+^TCRβ^+^TCRγδ^−^CD4^+^CD8α^−^) isolated from the CNS of naive *Bhlhe40*^fl/fl^ mice or immunized *Bhlhe40*^fl/fl^, *Cd4*-Cre^+^ *Bhlhe40*^fl/fl^, and *Il17a*-Cre^+^ *Bhlhe40*^fl/fl^ mice at day 14 after EAE induction. (**D**) Quantitation of CD4^+^ T cell cytokine production as described in (C) (pooled from three experiments, *n* = 3–10 per group). (**E**) Representative flow cytometry of GM-CSF^+^ and IL-10^+^ CD4^+^ T cells isolated from the CNS of immunized *Bhlhe40*^fl/fl^ and *Il17a*-Cre^+^ *Bhlhe40*^fl/fl^ mice at day 14 after EAE induction. (**F**) Quantitation of CD4^+^ T cell cytokine production as described in (E) (pooled from two experiments, *n* = 6–8 per group). (**G**) Representative flow cytometry of microglia (CD45^int^CD11b^+^) and infiltrating myeloid cell (CD45^high^CD11b^+^) activation (MHC class II^+^) from the indicated mice. (**H**) Quantitation of the data presented in (G) (pooled from seven experiments, *n* = 10–19 per group). Data are presented as mean ± SEM. Statistical significance was determined by a Mann–Whitney *U* test between the area under the curve for individual mice (A and B, left panels), Mann–Whitney *U* test (A and B, right panels), one-way ANOVA with a Tukey multiple comparison test (D and F), and an unpaired two-sided Student *t* test (F). *p < 0.05, **p < 0.01, ***p < 0.001, ****p < 0.0001. ns, not significant.

Because BHLHE40 was highly expressed in T_H_1/17 and ex-T_H_17 cells, we tested whether *Il17a*-Cre^+^ *Bhlhe40*^fl/fl^ mice would have an altered course of EAE. For comparison, we also induced EAE in *Cd4*-Cre^+^ *Bhlhe40*^fl/fl^ mice, which lack *Bhlhe40* expression in all T cells and which have previously been shown to be protected from EAE (34). As expected, *Cd4*-Cre^+^ *Bhlhe40*^fl/fl^ mice were highly protected during EAE compared with *Bhlhe40*^fl/fl^ controls (Fig. 2A). Notably, we found consistently reduced EAE severity upon *Il17a*-mediated deletion of *Bhlhe40*, although not to the same extent as *Cd4*-Cre–mediated deletion (Fig. 2B). We restimulated cells isolated from the CNS of naive mice and from immunized *Bhlhe40*^fl/fl^, *Cd4*-Cre^+^ *Bhlhe40*^fl/fl^, or *Il17a*-Cre^+^ *Bhlhe40*^fl/fl^ mice 14 d after immunization to examine cytokine production. In both *Cd4*-Cre^+^ *Bhlhe40*^fl/fl^ and *Il17a*-Cre^+^ *Bhlhe40*^fl/fl^ mice we found that the most striking reduction was in the IL-17A^−^IFN-γ^+^GM-CSF^+^ population, which was the most increased population in immunized *Bhlhe40*^fl/fl^ mice relative to naive mice (Fig. 2C, 2D). Based on our fate-mapping experiments, in *Il17a*-Cre^+^ *Bhlhe40*^fl/fl^ mice these cells likely represent ex-T_H_17 cells that had once produced IL-17A and therefore deleted *Bhlhe40*, reducing their potential for GM-CSF production. As BHLHE40 has been shown to negatively regulate IL-10 in CD4^+^ T cells (18), we examined IL-10 production in the context of *Il17a*-Cre–driven deletion of BHLHE40. At the peak of disease, *Il17a*-Cre^+^ *Bhlhe40*^fl/fl^ CD4^+^ T cells had no statistically significant difference in IL-10 production compared with *Bhlhe40*^fl/fl^ CD4^+^ T cells, although there was a trend toward greater IL-10 production upon BHLHE40 deletion (Fig. 2E, 2F). As expected, *Il17a*-Cre^+^ *Bhlhe40*^fl/fl^ CD4^+^ T cells had decreased total GM-CSF production. This suggests that the clinical protection seen in *Il17a*-Cre^+^ *Bhlhe40*^fl/fl^ mice is likely not due to local IL-10 production in the CNS.

CNS-infiltrating myeloid cells are the critical responders to GM-CSF produced by encephalitogenic CD4^+^ T cells (35, 36). We therefore examined the impact of altered cytokine production from BHLHE40-deficient CD4^+^ T cells on the myeloid cells present in the CNS during EAE. Upon immunization, there was infiltration of myeloid cells into the CNS of *Bhlhe40*^fl/fl^ control mice (Fig. 2G, 2H). These infiltrating myeloid cells, as well as CNS-resident microglia, upregulated MHC class II upon activation (Fig. 2G, 2H). In both immunized *Cd4*-Cre^+^ and *Il17a*-Cre^+^ *Bhlhe40*^fl/fl^ mice, there was a dramatic reduction in the number and activation of infiltrating myeloid cells and activation of microglia compared with immunized *Bhlhe40*^fl/fl^ mice (Fig. 2G, 2H). To test for the possibility of a cell-intrinsic role for BHLHE40 in infiltrating myeloid cells, we crossed *Bhlhe40*^fl/fl^ to *Lyz2*-Cre and actively induced EAE. We did not find a role for BHLHE40 in myeloid cells or in neutrophils using *S100a8*-mediated deletion (Supplemental Fig. 2). Overall, we show that altered cytokine production from BHLHE40-deficient CD4^+^ T cells resulted in a decrease in the number and activation of infiltrating myeloid cells.

### scRNA-seq identifies CNS leukocytes altered by BHLHE40 deficiency during EAE

**FIGURE 3.**
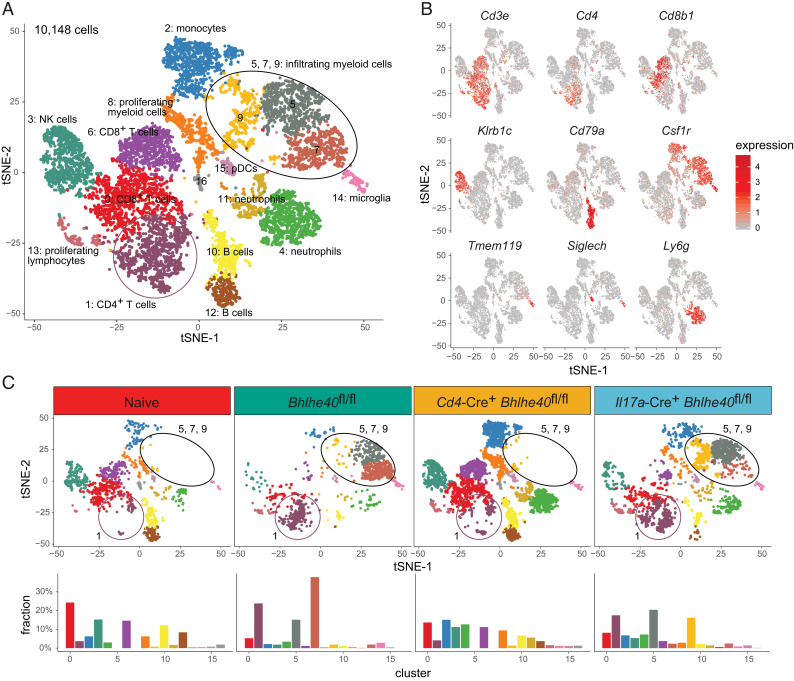
scRNA-seq of various *Bhlhe40* deletion models on day 14 of EAE. Live immune cells (CD45^+^7-AAD^−^) were isolated from the CNS of naive mice (*Bhlhe40*^fl/fl^, *n* = 4) or the CNS of actively immunized *Bhlhe40*^fl/fl^ mice (*n* = 3), *Cd4*-Cre^+^ *Bhlhe40*^fl/fl^ mice (*n* = 3), or *Il17a*-Cre^+^ *Bhlhe40*^fl/fl^ mice (*n* = 2) on day 14 after EAE induction. (**A**) tSNE plot of the combined scRNA-seq samples (total 10,148 cells). (**B**) Feature plots displaying cell type–identifying gene expression. (**C**) Top, tSNE plots of immune cell clusters split by sample: naive (1293 cells), immunized *Bhlhe40*^fl/fl^ (1615 cells), *Cd4*-Cre^+^ *Bhlhe40*^fl/fl^ (4414 cells), and *Il17a*-Cre^+^ *Bhlhe40*^fl/fl^ (2826 cells). Bottom, Percentage of each cluster present in each of the indicated samples. 7-AAD, 7-aminoactinomycin D.

To further probe how deletion of BHLHE40 in T cells or IL-17A–producing cells impacts on the immune cells present in the CNS during EAE, we performed scRNA-seq. Immune cells (CD45^+^) were sorted from the CNS of naive *Bhlhe40*^fl/fl^ controls or from immunized *Bhlhe40*^fl/fl^, *Cd4*-Cre^+^ *Bhlhe40*^fl/fl^, or *Il17a*-Cre^+^ *Bhlhe40*^fl/fl^ mice on day 14 of EAE. After dimensionality reduction of the scRNA-seq data, immune cells partitioned into 17 unique clusters (Fig. 3A). Clusters were identified based on gene expression of common lineage-specific markers (Fig. 3B, Supplemental Fig. 3A). Immunization of *Bhlhe40*^fl/fl^ mice resulted in an increased fraction of CD4^+^ T cells (cluster 1) and myeloid cells (clusters 5 and 7) infiltrating into the CNS compared with naive mice (Fig. 3C, Supplemental Fig. 3B). This increase was not seen in the CNS of immunized *Cd4*-Cre^+^ *Bhlhe40*^fl/fl^ mice, which largely resembled the naive CNS with the exception of an increased frequency of monocytes (cluster 2) and neutrophils (clusters 4 and 11) (Fig. 3C). The CNS of immunized *Il17a*-Cre^+^ *Bhlhe40*^fl/fl^ mice largely resembled those of immunized *Bhlhe40*^fl/fl^ mice, with a high percentage of CD4^+^ T cells (cluster 1) and infiltrating myeloid cells (the collection of clusters 5, 7, and 9) (Fig. 3C, Supplemental Fig. 3B). Based on our sorting strategy using CD45 expression, few microglia (cluster 14) were sequenced from our various samples. In general, microglia resembled myeloid cluster 7 in terms of gene expression based on their proximity on the tSNE plot, but due to their low abundance in individual samples, comparisons between genotypes were not made.

To determine the different pathways upregulated or downregulated in CD4^+^ T cells from immunized *Bhlhe40*^fl/fl^ and *Il17a*-Cre^+^ *Bhlhe40*^fl/fl^ mice, we cross-referenced the list of top differentially expressed genes to gene signatures within the MSigDB. CD4^+^ T cells from the immunized *Bhlhe40*^fl/fl^ CNS upregulated genes in pathways involved in the innate immune system and complement (Supplemental Fig. 3C). The pathways most enriched in CD4^+^ T cells from immunized *Il17a*-Cre^+^ *Bhlhe40*^fl/fl^ CNS included both type 1 and type 2 IFN responses (Supplemental Fig. 3D).

**FIGURE 4.**
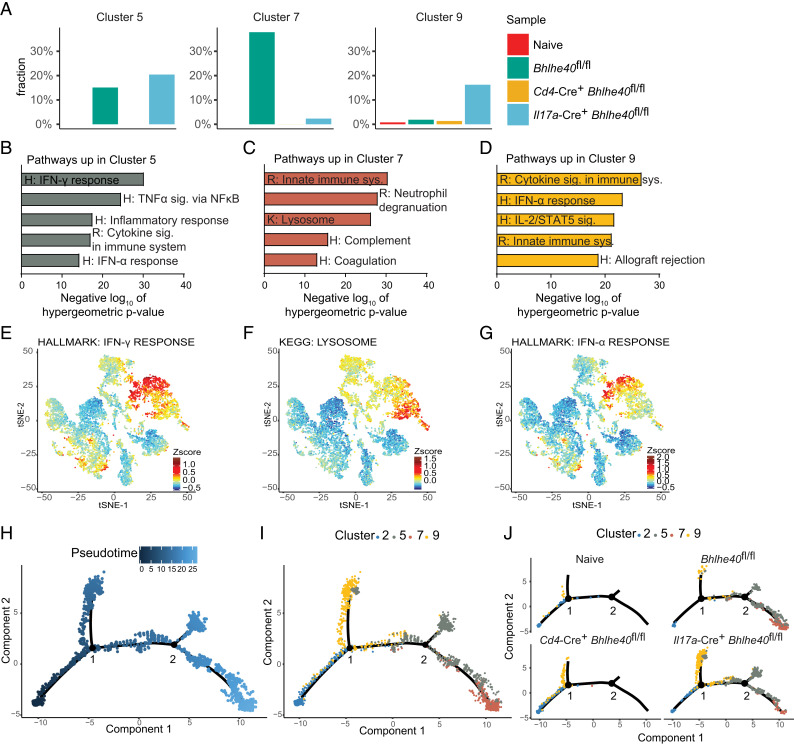
Differences in myeloid cell gene expression in *Il17a*-Cre^+^ *Bhlhe40*^fl/fl^ mice. (**A**) Fraction of infiltrating myeloid cells (clusters 5, 7, and 9) in the CNS of naive *Bhlhe40*^fl/fl^ mice or immunized *Bhlhe40*^fl/fl^, *Cd4*-Cre^+^ *Bhlhe40*^fl/fl^, and *Il17a*-Cre^+^ *Bhlhe40*^fl/fl^ mice at day 14 after EAE induction. (**B**–**D**) Differentially expressed genes (adjusted *p* value >0.05 and log_2_ fold change ≥0.35) between clusters 5, 7, and 9 were cross-referenced to hallmark (H), Reactome (R), and KEGG (K) gene sets in the MSigDB for enriched pathways. (**E**–**G**) Pathways of interest for clusters (E) 5, (F) 7, and (G) 9 mapped onto the grouped tSNE plot. (**H**) Pseudotime plot generated by Monocle 2 from the scRNA-seq myeloid clusters 2, 5, 7, and 9. Branch points 1 and 2 are indicated. (**I**) Myeloid clusters 2, 5, 7, and 9 mapped onto the Monocle 2 pseudotime plot. (**J**) Myeloid clusters 2, 5, 7, and 9 mapped onto the Monocle 2 pseudotime plot and separated by sample.

We further examined several clusters of infiltrating myeloid cells (clusters 5, 7, and 9). Cluster 5 was present in immunized *Bhlhe40*^fl/fl^ and *Il17a*-Cre^+^ *Bhlhe40*^fl/fl^ CNS in roughly equal proportions (Fig. 4A). Cluster 7 was dramatically higher in immunized *Bhlhe40*^fl/fl^ CNS, compared with its near absence in the other samples. Similarly, cluster 9 was largely specific for the *Il17a*-Cre^+^ *Bhlhe40*^fl/fl^ CNS. Cluster 5 was enriched in inflammatory pathways, such as IFN-γ response, TNF-α signaling, inflammatory response, and cytokine signaling (Fig. 4B, 4E). Cluster 7 appeared to be a highly phagocytic population, with enrichment in pathways such as neutrophil degranulation, lysosome, and complement (Fig. 4C, 4F). Cluster 9 was enriched in cytokine signaling and type 1 IFN responses (Fig. 4D, 4G), similar to the CD4^+^ T cells from immunized *Il17a*-Cre^+^ *Bhlhe40*^fl/fl^ CNS. The emergence of a type 1 IFN signature in cluster 9, which was almost exclusively present in the *Il17a*-Cre^+^ *Bhlhe40*^fl/fl^ CNS, and the CD4^+^ T cells from this same sample was notable. In summary, deletion of *Bhlhe40* from IL-17A–producing cells resulted in CNS-infiltrating myeloid cells with altered phenotypes during EAE.

To examine the potential for developmental relationships between the myeloid clusters, we employed the Monocle 2 algorithm (32, 33) to assign a pseudotime trajectory to the myeloid cells in clusters 2, 5, 7, and 9 (Fig. 4H). We included cluster 2 (monocytes) in this analysis, as these cells likely represent the precursor population that gives rise to the other myeloid clusters (36–39). When the clusters were mapped onto the pseudotime plot, monocytes (cluster 2) were predicted to be the precursor that progresses through one of two differentiation pathways (Fig. 4I, 4J). The first branch point (branch point 1) was where most of cluster 9 resided, and the second branch point (branch point 2) involved a differentiation from cluster 5 to cluster 7. Part of cluster 5 appeared to be a distinct cell fate, while the rest of cluster 5 appeared mostly in the transition between clusters 9 and 7. This is consistent with the fact that some of the enriched pathways present in cluster 5 are also shared pathways with cluster 9 (Fig. 4B, 4D). It appeared that cluster 9, an IFN-responsive population, is a cell fate that was largely unlocked by the absence of *Bhlhe40* from IL-17A–producing cells. Overall, scRNA-seq revealed regulation of myeloid cell gene expression by pathogenic CD4^+^ T cells in a BHLHE40-dependent manner.

## Discussion

We sought to interrogate the expression of and role for BHLHE40 in T_H_17 cells and T_H_17-cell derived populations (T_H_1/17 and ex-T_H_17 cells) during active EAE. Using our dual reporter system we demonstrate that after EAE induction, BHLHE40 is expressed in both T_H_1/17 and ex-T_H_17 cells and that BHLHE40 expression correlates with GM-CSF production. Deletion of BHLHE40 either in all T cells (*Cd4*-Cre) or in T_H_17 cells (*Il17a*-Cre) was protective from clinical EAE disease. The CNS of immunized *Il17a*-Cre^+^ *Bhlhe40*^fl/fl^ mice contained fewer IL-17A^−^IFN-γ^+^GM-CSF^+^CD4^+^ T cells compared with immunized *Bhlhe40*^fl/fl^ control mice, suggesting that BHLHE40 regulates pathogenicity at a step subsequent to T_H_17 differentiation, likely as these cells convert into ex-T_H_17 cells. As a consequence of altered CD4^+^ T cell cytokine production, there were fewer and less activated infiltrating myeloid cells in the CNS at the peak of EAE clinical disease of *Il17a*-Cre^+^ *Bhlhe40*^fl/fl^ mice.

Using scRNA-seq we show how populations of immune cells in the CNS change with the presence or absence of BHLHE40 in our different deletion systems. The *Cd4*-Cre^+^ *Bhlhe40*^fl/fl^ CNS largely resembled the CNS of naive mice, with low percentages of pathogenic CD4^+^ T cells and few infiltrating myeloid cells compared with the increases seen in the CNS of immunized *Bhlhe40*^fl/fl^ and *Il17a*-Cre^+^ *Bhlhe40*^fl/fl^ mice. Both CD4^+^ T cells (cluster 1) and myeloid cluster 9 (abundant in the *Il17a*-Cre^+^ *Bhlhe40*^fl/fl^ CNS) were enriched for IFN response pathways, highlighting a potential mechanism of protection upon deletion of BHLHE40 in T_H_17 cells, potentially downstream of decreased GM-CSF and IFN-γ production by BHLHE40-deficient CD4^+^ T cells.

GM-CSF production from encephalitogenic T cells is essential for infiltrating myeloid cell activation in the CNS during EAE (6, 35, 36, 38, 40, 41). We hypothesize that in the setting of *Cd4*-Cre–mediated deletion of *Bhlhe40*, there is not enough GM-CSF produced from CD4^+^ T cells to differentiate infiltrating monocytes in the CNS, potentially explaining why this scRNA-seq sample lacks monocyte-derived cells (clusters 5, 7, and 9). In the case of *Il17a*-Cre–mediated deletion of *Bhlhe40*, there may still be enough GM-CSF production from unaffected T_H_1 cells to activate infiltrating monocytes, allowing these cells to differentiate into unique cell fates. The loss of *Bhlhe40* in T_H_1/17 and ex-T_H_17 cells in *Il17a*-Cre^+^ *Bhlhe40*^fl/fl^ mice allows for a new type I IFN–responsive myeloid cell fate to emerge (cluster 9). It is interesting that both the protective effects of endogenous type I IFNs and the pathogenic effects of GM-CSF act through monocyte-derived cells that directly respond to these cytokines during active EAE (42). GM-CSF may directly inhibit a monocyte-derived cell’s ability to effectively respond to type I IFNs, which has been suggested in vitro (43) and recently in vivo during EAE (36).

Additionally, it has been suggested that one mechanism of protection by type I IFNs is to limit IL-1β production from CNS-infiltrating monocytes, resulting in less IL-1β–dependent GM-CSF production from CD4^+^ T cells (44). BHLHE40 deficiency in IL-17A–producing CD4^+^ T cells could disrupt the normal GM-CSF/IL-1β–dependent positive amplification loops shown to take place during EAE (20, 36, 44–46). A more comprehensive understanding of cytokine production by autoreactive CD4^+^ T cells and cytokine feedback loops active in infiltrating monocytes in EAE could inform future therapeutic discovery efforts in multiple sclerosis. Recombinant IFN-β was the first Food and Drug Administration–approved therapy for multiple sclerosis, although its clinical efficacy has been surpassed by other therapeutics. Perhaps a dual approach of altering myeloid cells’ responsiveness to pathogenic signals (GM-CSF) and enhancing their responsiveness to protective signals (type I IFNs) may be worthy of future investigation.

## Supplementary Material

Supplemental Figures 1 (PDF)

Supplemental Table 1 (XLS)
