# Supplementary material for: BHLHE40 Mediates Cross-Talk between Pathogenic TH17 Cells and Myeloid Cells during Experimental Autoimmune Encephalomyelitis
Source: Immunohorizons. 2023 Nov 7;7(11):737–46. doi: 10.4049/immunohorizons.2300042 (PMC10695412; doi:10.4049/immunohorizons.2300042)
Supplement: Supplemental Figures 1 (PDF) [file IH_2300042_Supplemental_Figures_1.pdf]

Supplemental Figure 1

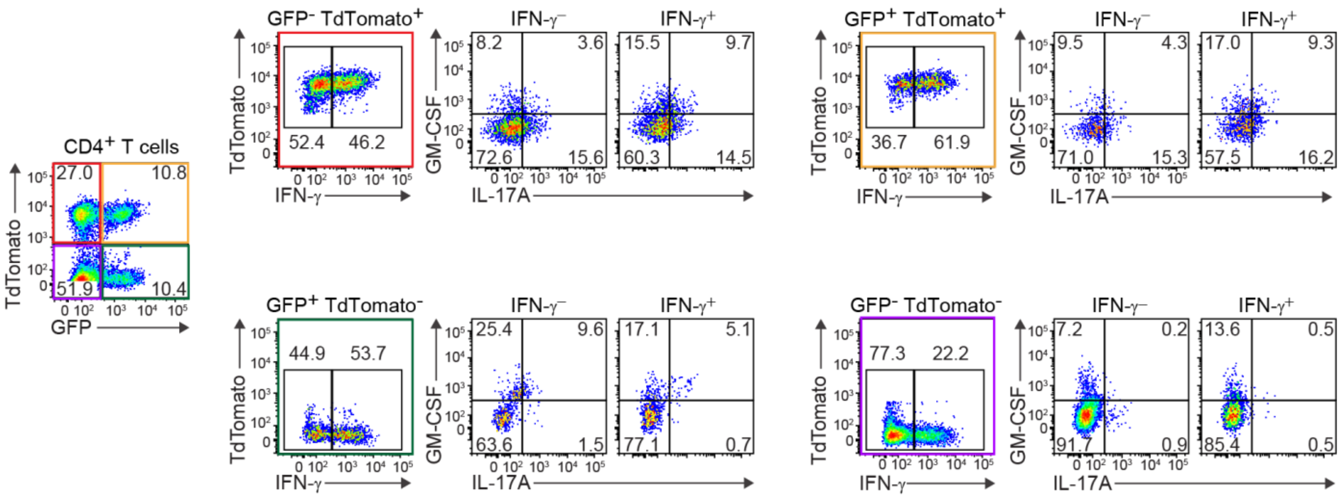

Supplemental Figure 1: Representative cytokine production from *Il17a*-Cre *Rosa26*-TdTomato *Bhlhe40*^{GFP} reporter CD4^{+} T cells. Representative intracellular cytokine staining of *Il17a*-Cre *Rosa26*-TdTomato *Bhlhe40*^{GFP} reporter CNS CD4^{+} T cells (CD45.2^{+} TCRβ^{+} TCRγδ^{-} CD8α^{-} CD4^{+}) on day 14 of EAE. Gated quadrants are indicated by colored boxes.

Supplemental Figure 2

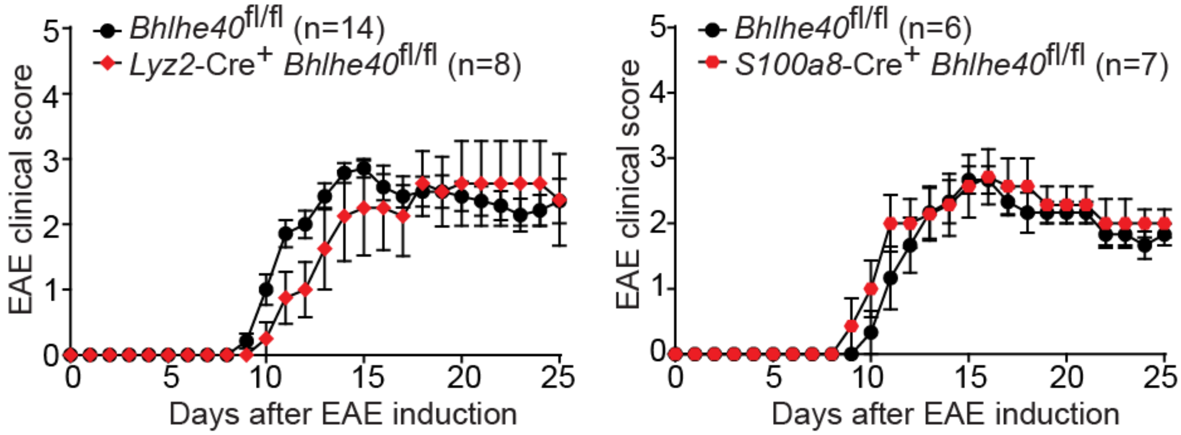

Supplemental Figure 2: No cell-intrinsic role for BHLHE40 in myeloid cells or neutrophils during EAE. Clinical EAE scores from immunized *Lyz2*-Cre^{-} *Bhlhe40*$^{fl/fl}$ or *Lyz2*-Cre^{+} *Bhlhe40*$^{fl/fl}$ mice (pooled from two experiments, *n* = 8-14 per group) and *S100a8*-Cre^{-} *Bhlhe40*$^{fl/fl}$ or *S100a8*-Cre^{+} *Bhlhe40*$^{fl/fl}$ mice (one experiment, *n* = 6-7 per group).

Supplemental Figure 3

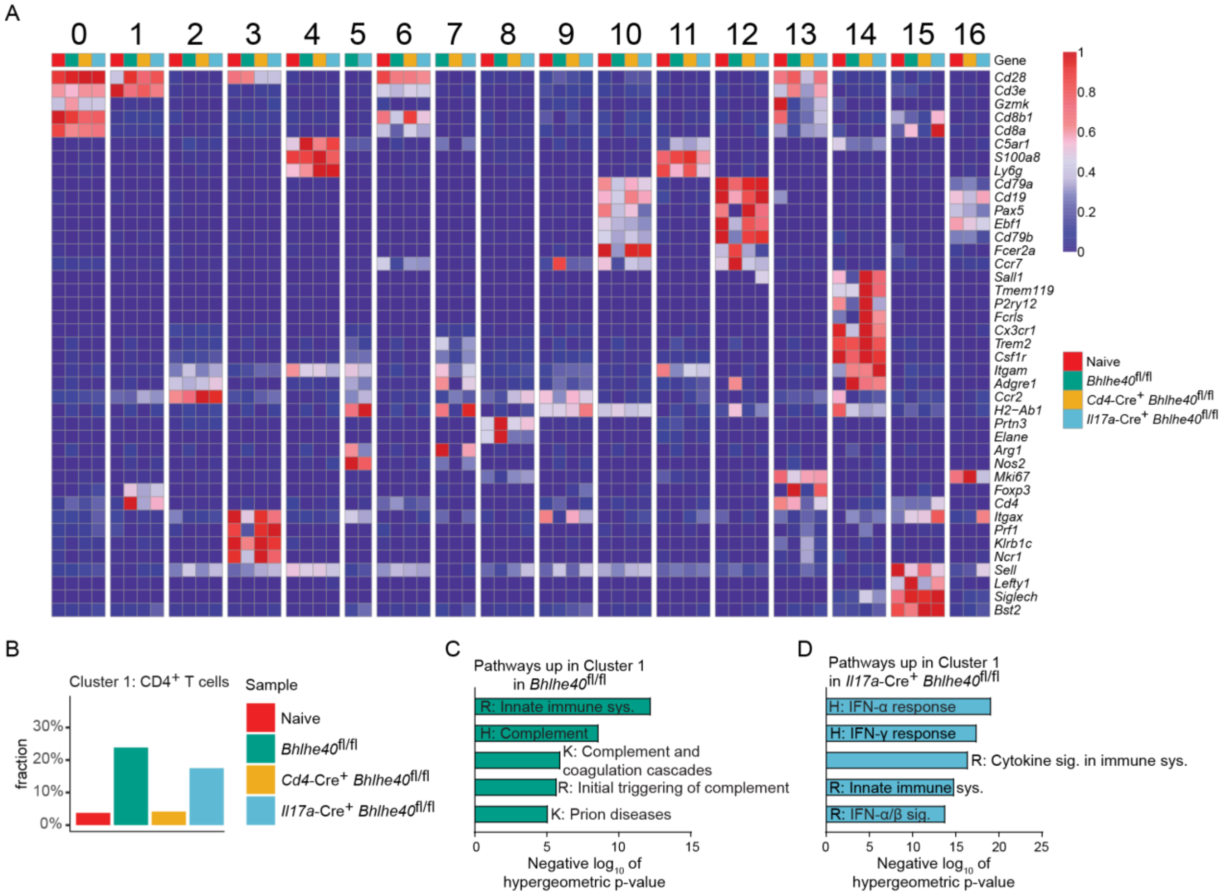

Supplemental Figure 3: scRNA-seq cell type identifying genes and characterization of CD4$^+$ T cells from *Bhlhe40*$^{fl/fl}$ and *Il17a*-Cre$^+$ *Bhlhe40*$^{fl/fl}$ mice. (A) Gene expression heatmap of cell type identifying genes separated by cluster and by sample from scRNA-seq data from the CNS of naïve *Bhlhe40*$^{fl/fl}$ mice or immunized *Bhlhe40*$^{fl/fl}$, *Cd4*-Cre$^+$ *Bhlhe40*$^{fl/fl}$, and *Il17a*-Cre$^+$ *Bhlhe40*$^{fl/fl}$ mice at day 14 post EAE induction. **(B)** Fraction of CD4$^+$ T cells (cluster 1) in the CNS of naïve *Bhlhe40*$^{fl/fl}$ mice or immunized *Bhlhe40*$^{fl/fl}$, *Cd4*-Cre$^+$ *Bhlhe40*$^{fl/fl}$, and *Il17a*-Cre$^+$ *Bhlhe40*$^{fl/fl}$ mice at day 14 post EAE induction **(C and D)** Differentially expressed genes (adjusted p-value >0.05 and $\log_2$ fold-change $\geq$0.35) were cross-referenced to Hallmark (H), Reactome (R), and KEGG (K) gene sets in the MSigDB for enriched pathways between immunized *Bhlhe40*$^{fl/fl}$ and *Il17a*-Cre$^+$ *Bhlhe40*$^{fl/fl}$ cluster 1 cells.
